# Ultrasound-modulated optical glucose sensing using a 1645 nm laser

**DOI:** 10.1038/s41598-020-70305-6

**Published:** 2020-08-07

**Authors:** Eun-Yeong Park, Jinwoo Baik, Hyojin Kim, Sung-Min Park, Chulhong Kim

**Affiliations:** Departments of Electrical Engineering, Creative IT Engineering, and Mechanical Engineering, and School of Interdisciplinary Bioscience and Bioengineering, Pohang University of Science and Technology (POSTECH), 77 Cheongam-Ro, Nam-Gu, Pohang, Gyeongbuk 37673 Republic of Korea

**Keywords:** Diabetes, Imaging and sensing

## Abstract

Regular and frequent blood glucose monitoring is vital in managing diabetes treatment plans and preventing severe complications. Because current invasive techniques impede patient compliance and are not infection-free, many noninvasive methods have been proposed. Among them, optical methods have drawn much attention for their rich optical contrast, but their resolution is degraded in deep tissue. Here, we present an ultrasound-modulated optical sensing (UOS) technique to noninvasively monitor glucose that uses an infrared laser (1645 nm) and a single-element focused ultrasound transducer. Focused ultrasound waves can acoustically localize diffused photons in scattering media, and thus optical contrast can be represented with much enhanced spatial resolution. To maximize the signal-to-noise ratio, we compared the modulation depths of UOS signals in both continuous and burst ultrasound transmission modes. Finally, UOS measurements of various glucose concentrations are presented and compared with those acquired in phantoms with a conventional diffuse optical sensing method. The UOS measurements in a 20 mm thick tissue-mimicking phantom show 26.6% accuracy in terms of mean absolute relative difference (MARD), which indicates the great potential of the proposed technique as a noninvasive glucose sensor.

## Introduction

Diabetes mellitus is a chronic metabolic disorder that causes high blood sugar (glucose) levels due to either insufficient production of insulin or inadequate cellular response to the produced insulin^1^. Diabetes can lead to serious complications, such as cardiovascular disease, stroke, blindness, chronic renal failure, neuropathy, or even death^2–4^. As of 2019, it is estimated that 463 million people are suffering from diabetes worldwide, and this population is projected to reach 700 million by 2045^5^. In 2012, the total cost of diagnosed diabetes in the USA was estimated to be $245 billion, and in 2013 over five million people between 20–79 years of age were estimated to die from diabetes-associated causes worldwide^6,7^. Although there is no known cure for the disease, diabetic patients can minimize complications through timely and constant care and treatment in conjunction with regular blood glucose monitoring.

Current glucose sensors require either drawing a drop of blood by finger-pricking or inserting the sensor under the skin. The repetitive use of these invasive devices causes pain and can lead to infection, tissue damage, or reduced patient compliance. Accordingly, noninvasive glucose sensors have been increasingly sought in recent decades^8–12^. Most noninvasive sensors are based on electromagnetic (EM) waves, especially within optical wavelengths, because these waves interact richly with various components of biological tissues. Raman spectroscopy, using near-infrared (NIR) and mid-infrared (MIR) illumination, detects the Raman shift from inelastic scattering by glucose molecules. It provides high specificity and is relatively robust to water and temperature changes, but requires a long collection time^13,14^. Optical coherent tomography (OCT) captures changes in the refractive index of interstitial fluid with varying glucose concentrations and offers a high signal-to-noise ratio (SNR) and resolution^15^. NIR and MIR spectroscopy, which use photon energy within the fundamental and overtone vibration bands of glucose molecules^16,17^, can provide absorption contrast with relatively good specificity^18–20^. Although these optical sensing methods have great potential as noninvasive glucose sensors, they still are hindered by their limited penetration depth and degraded spatial resolution in deep tissue. With these aspects, their sensing target is usually restricted to tears or interstitial fluid, which might have different glucose levels from the blood especially when the blood glucose is rapidly changing such as postprandial state^21,22^.

Like photoacoustic imaging, ultrasound-modulated optical tomography (UOT) is a noninvasive biomedical imaging technique that visualizes optical characteristics with a high ultrasonic spatial resolution^23–31^. UOT acoustically localizes scattered light by irradiating both coherent laser light and focused ultrasound waves in optically scattering media. Scattering media insonified by focused ultrasound exhibit changes in their local scatterer displacement and refractive index, and accordingly modulate photons traversing the acoustic focal region^32–35^. By exploiting the strengths of both optical and ultrasound imaging, UOT can overcome the limitations of pure optical imaging and provide imaging at depth in the diffusive regime with enhanced spatial resolution^36–42^.

In this paper, we propose an ultrasound-modulated optical sensing (UOS) system for noninvasive glucose measurement that uses an infrared laser and a single-element focused ultrasound transducer. To achieve high optical absorption contrast, we use a 1645 nm laser, based on the measured glucose absorbance. The modulation efficiencies of continuous ultrasound waves and ultrasound bursts are experimentally compared while maintaining the ultrasound intensity under the diagnostic ultrasound safety limit. Finally, using a vessel-mimicking phantom, we explore the dependency of UOS signals on glucose concentration by acquiring UOS signals from various glucose concentrations.

## Results

### Ultrasound-modulated optical glucose sensing system

**Figure 1:**
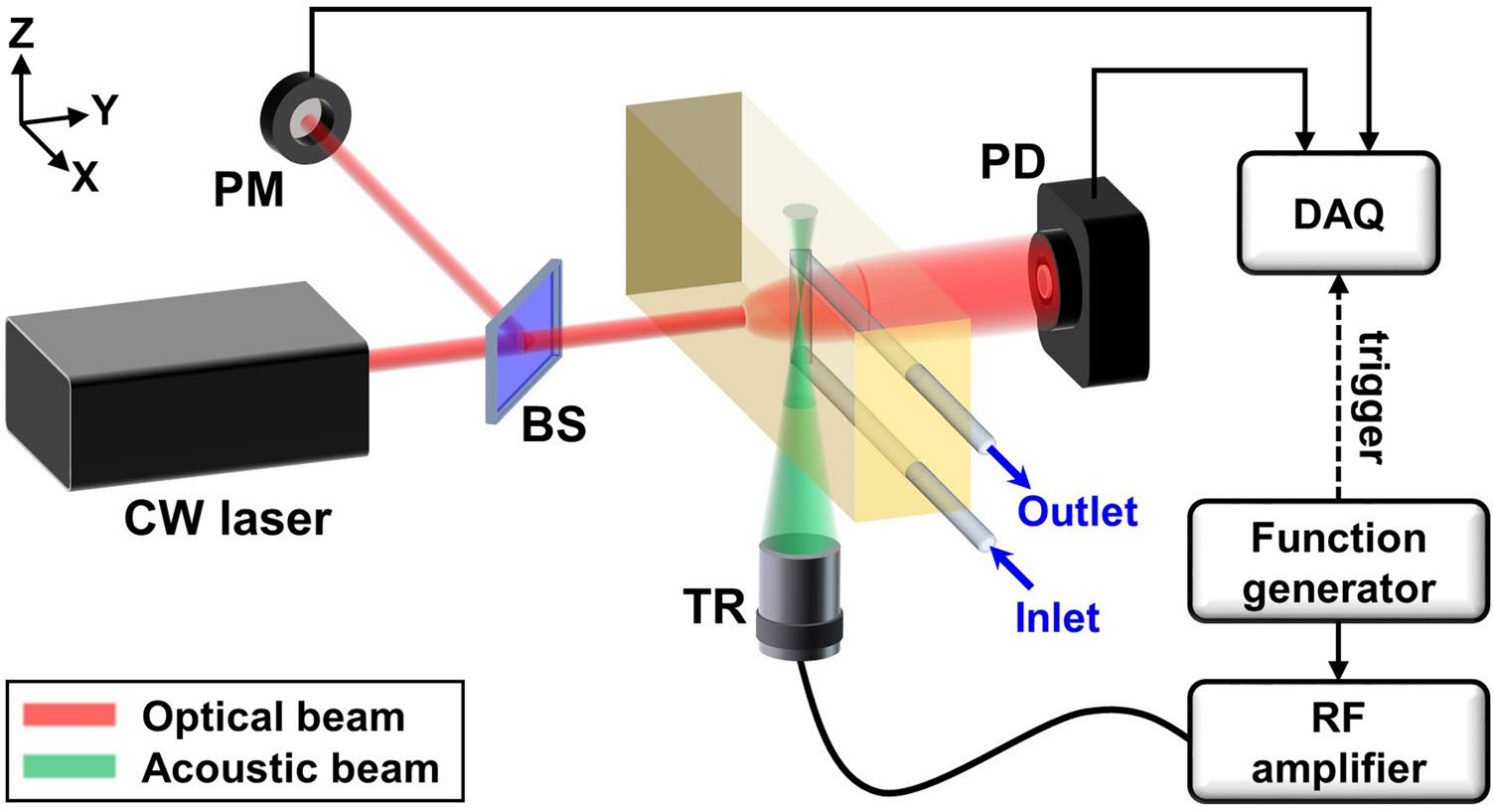
Schematic of an ultrasound-modulated optical sensing system. *CW* continuous wave, *BS* beam splitter, *PM* power meter, *TR* ultrasound transducer, *PD* photodiode, *RF* radio frequency,*DAQ* data acquisition system.

Figure 1 shows a schematic and photograph of the UOS system. A diode-pumped solid state laser (MIL-N-1645, CNI Laser, China) with a wavelength of 1645 nm and a coherence length of > 10 cm irradiated a tissue-mimicking phantom. The laser fluence on the target surface was about 40 mJ/cm^2^ for 5 ms per each measurement, which is far below the ANSI safety standard of 1,489 mJ/cm^2^ for 1645 nm laser exposure on skin^43^. Ultrasound waves were generated and focused into the phantom by an ultrasound transducer (KPS100-1-P38, The Ultran Group, USA) with a central frequency of 1 MHz, an active diameter of 25 mm, and a focal length of 38 mm. The ultrasound and optical propagation axes were set perpendicular to each other, and the ultrasound focus was located at the intersection of the two paths. A function generator (33220A, Agilent Technologies, USA) produced 1 MHz sinusoidal waveforms in continuous wave (CW) or burst mode, which were then amplified by an RF power amplifier (325LA, Electronics and Innovation, USA) with a fixed gain of 50 dB to drive the ultrasound transducer. The light transmitted through the phantom was collected by a photo diode (PDA10D2, Thorlabs, USA; ø1.0 mm photosensitive area; 900–2,600 nm spectral response range). The corresponding light intensity was recorded by a data acquisition system (DAQ; ATS9350, Alazar Technologies, Canada; 12 bit resolution; up to 500 MS/s sampling rate; ± 4 V input range). The data acquisition was synchronized with the ultrasound firing using the trigger signal from the function generator. A UV fused silica beam splitter (BSN12R, Thorlabs, USA; 10:90 reflectance-to-transmission split ratio) split the input light, which was collected by a power meter (G8931-20, Hamamatsu Photonics, Japan; ø0.2 mm photosensitive area; 950–1,700 nm spectral response range) to normalize the light fluence.

### Optical properties of the in vitro phantom

We evaluated the optical properties of the in vitro phantom to validate its similarity to biological tissues. The background of the phantom was composed of 0.2 g/mL gelatin, 0.1 mg/mL TiO_2_ powder (232033, Sigma-Aldrich, USA), and heavy water (151882, Sigma-Aldrich, USA), with dimensions of 60 mm (X) $\times$ 20 mm (Y) $\times$ 40 mm (Z)^44^. The measured effective attenuation coefficient was 3.1 cm^-1^ at 1645 nm. To prove the similarity between blood vessel and the tubing used for mimicking blood vessel, we excised carotid artery of a rat (female, 8 weeks) and measured the attenuation of light in the vessel while sandwiching it between two sheets of cover glass (Cover Slips, Duran Group, Germany; 24 mm $\times$ 50 mm, 0.16–0.19 mm thickness). The size of the excised blood vessel was about 7.5 mm $\times$ 2.5 mm. All of the animal procedures were conducted in accordance with the Pohang University of Science and Technology (POSTECH) Institutional Animal Care and Use Committee (IACUC) protocols. We also measured the attenuation of light in the tubing in the same manner. The transmitted laser power after the cover glass, the cover glass embedding the blood vessel, and the cover glass embedding the tubing were 93.9%, 87.2%, and 86.1%, respectively, which shows their similarity in optical attenuation.

### Optical absorption contrast of glucose solution

**Figure 2:**
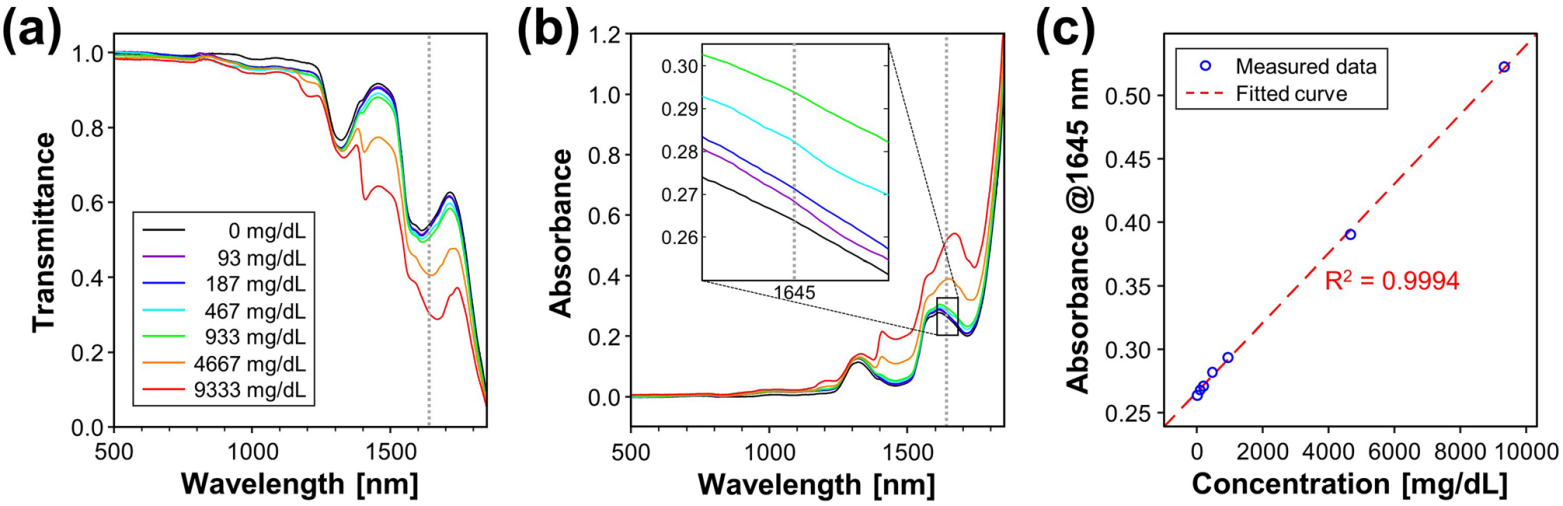
Optical transmittance (**a**) and absorbance (**b**) spectra of glucose solutions and heavy water. The dotted lines in gray represent the wavelength of the laser (1,645 nm) in the UOS system. (**c**) Absorbance values at 1,645 nm and the fitted regression line. UOS, ultrasound-modulated optical sensing.

To verify the optical absorption contrast of glucose solution, we separately obtained the absorbance spectra of various concentrations of glucose solutions and heavy water. Figure 2a and b show the optical transmittance and the absorbance of the glucose solutions and heavy water. Here, 0 mg/dL denotes the heavy water. The absorbance beyond 1,850 nm is omitted because converted absorbance values in a range where the transmittance is close to zero have low reliability. At 1645 nm, the optical wavelength used in the developed UOS system (gray dotted lines in Fig. 2a,b), the absorbance distinctly increases with concentration of glucose solution while having relatively high water transmittance of 54%, which could achieve a high glucose absorption contrast over water. Figure 2c represents the absorbance values of the glucose solutions at 1645 nm and the fitted linear regression line. The measured absorbance at 1645 nm exhibits direct linear response to the concentration of glucose solution with an R-squared value of 0.9994, which demonstrates the capability for glucose sensing using the proposed optical wavelength in water-based medium such as biological tissues.

### Modulation depth enhancement with acoustic bursts

The international standard IEC 60601-2-37 and the FDA^45,46^ stipulate that for medical diagnostic ultrasound systems in non-ophthalmic uses the thermal and mechanical indices should be less than 6.0 and 1.9, respectively. IEC 62,359 defines the mechanical index (MI) and the soft-tissue thermal index (TIS) as

1 $$MI= \underset{z}{max}\left\{\frac{{p}_{r.3}\left(z\right)\times {{f}_{awf}}^{-1/2}}{{C}_{MI}}\right\},$$

2 $$TIS= \underset{z}{max}\left\{\underset{}{min}\left[\frac{{W}_{.3}(z)\times {f}_{awf}}{{C}_{TIS1}},\frac{{I}_{SPTA.3}(z)\times {f}_{awf}}{{C}_{TIS2}}\right]\right\},$$

where $z$ is the distance from the external transducer aperture to the point of interest; ${p}_{r.3}\left(z\right)$, ${W}_{.3}(z)$, and ${I}_{SPTA.3}(z)$ are the attenuated peak rarefaction pressure, power, and spatial-peak temporal-average intensity at a depth $z$ with an acoustic attenuation coefficient of 0.3 dB/cm/MHz; ${f}_{awf}$ is the acoustic working frequency; and ${C}_{MI}$, ${C}_{TIS1}$, and ${C}_{TIS2}$ are constants of 1 MPa·MHz^-1/2^, 210 mW·MHz, and 210 mW·cm^−2^·MHz, respectively^47–49^. By definition, when applied the same driving voltage, MIs in CW and burst modes would be the same and TIS in burst mode would be lower in proportion to the duty cycle $\alpha$ ($0\le \alpha \le 1$) than TIS in CW mode.

To find the maximum driving voltage satisfying both the MI and TIS safety limits, we measured MI and TIS values by applying various driving voltages to the transducer in CW and burst modes, using an acoustic intensity measurement system (AIMS III, Onda, USA). In both CW and burst modes, 1 MHz sinusoidal waves were applied, and in burst mode, 10 μs bursts with a repetition period of 100 μs, i.e. a duty cycle of 0.1, were applied. Table 1 shows the maximum driving voltages within MI (V_MAX, MI_), and TIS (V_MAX, TIS_). The maximum allowable driving voltage within the MI limit for CW mode and that for burst mode are almost the same, whereas the maximum allowable driving voltage within the TIS limit for burst mode is about 3.3 times higher than that for CW mode, as expected^50,51^. Note that the theoretical value of the ratio between V_MAX, TIS_ in burst mode with a duty cycle of 0.1 and that in CW mode is $1/\sqrt{0.1}\approx 3.2$. The maximum driving voltage values, V_MAX_, satisfying both the MI and TIS safety limits and given by V_MAX_ = min[V_MAX, MI_, V_MAX, TIS_], are 20.6 V_pp_ and 68.6 V_pp_ in CW and burst modes, respectively.

**Table 1:** The measured acoustic output parameters and corresponding modulation depths in CW and burst modes.

	CW	Burst
V_MAX, MI_	72.7 V_pp_	73.9 V_pp_
V_MAX, TIS_	20.6 V_pp_	68.6 V_pp_
V_MAX_	20.6 V_pp_	68.6 V_pp_
MI @V_MAX_	0.56	1.76
TIS @V_MAX_	5.92	5.93
M_d_ @V_MAX_	0.062	0.154

V_MAX, MI_ and V_MAX, TIS_ represent the maximum driving voltages applied to the transducer within MI and TIS safety limits, respectively. V_MAX_ is the maximum driving voltage satisfying both MI and TIS safety limit.

*CW* continuous wave, *MI* mechanical index, *TIS* thermal index in soft tissue, *M*_*d*_ modulation depth.

**Figure 3:**
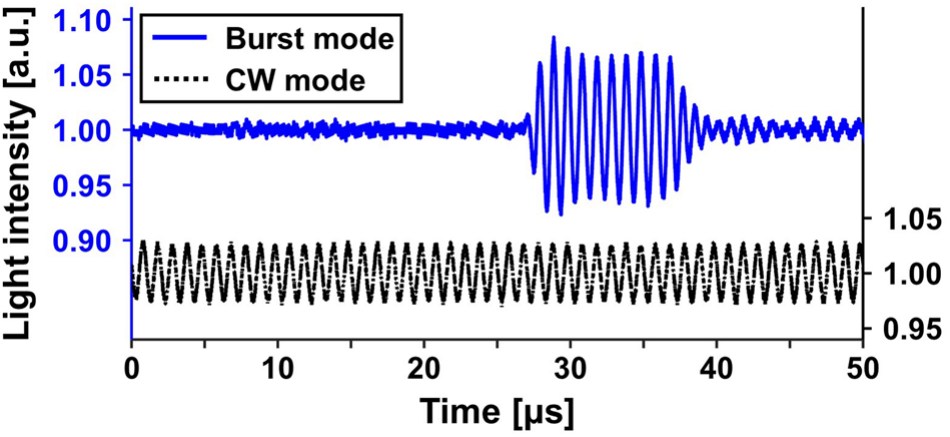
Time-resolved UOS signals from a 20-mm-thick tissue-mimicking phantom using ultrasound modulation in CW and burst modes. *UOS* ultrasound-modulated optical sensing, *CW* continuous wave.

Using the maximum allowable driving voltages within the diagnostic ultrasound safety limits (MI < 1.9 and TIS < 6.0), we obtained UOS signals in CW and burst modes from a 20-mm-thick tissue-mimicking phantom. Figure 3 shows the resultant time-resolved UOS signals in CW and burst modes. The light intensity is modulated at the ultrasound frequency during the entire time window in CW mode, and during the duty cycle (0.1 for a 100 μs period = 10 μs) in burst mode. The modulated light starts to appear at about 27 μs in burst mode, which matches well with the ultrasound time of flight to the crossing point of the light and ultrasound paths at the focal length (38 mm) of the ultrasound transducer, which can be converted to 25.3 μs assuming the speed of sound is 1,500 m/s. The modulation depth, defined as the ratio of the ultrasound-modulated light intensity to the unmodulated light intensity, was obtained as 0.062 and 0.154 in CW and burst modes, respectively (Table 1). As expected, we could achieve about 2.5 times greater modulation depth in burst mode than in CW mode. This greater depth was possible because we could apply much higher acoustic pressure to the sample in burst mode, since the sample could cool during the off cycle, whereas the sample remained heated in CW mode.

### In vitro vessel-mimicking phantom measurements

**Figure 4:**
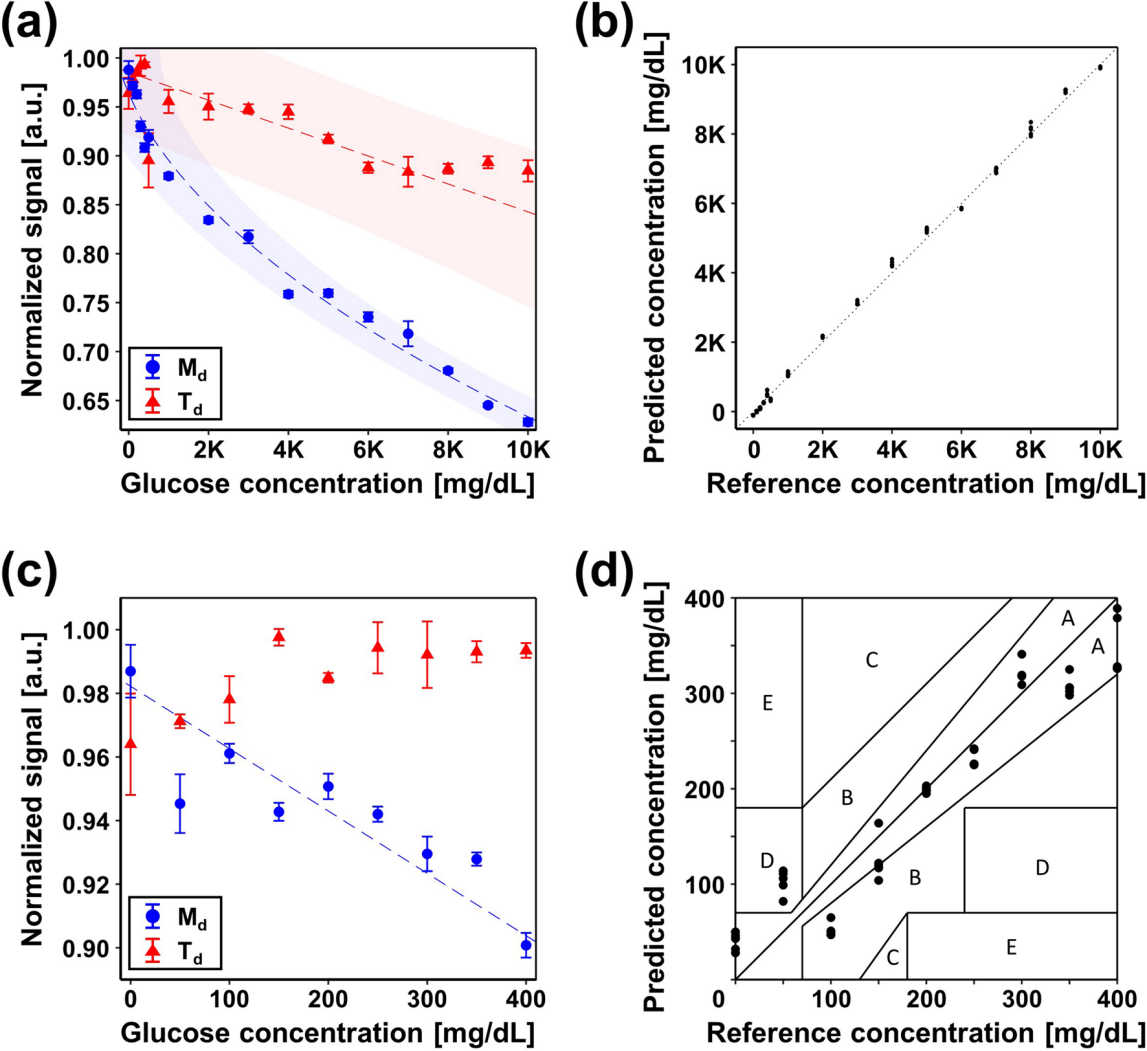
(**a**) Comparison of modulation depth and diffuse transmittance of various glucose concentrations. The error bars represent ± 1 standard deviation. The dashed lines and the shaded areas represent fitted curves of feature value to glucose concentrations and their 95% confidence interval. (**b**) Predicted glucose concentration obtained from UOS measurements of glucose solutions. (**c**) Close-up of measurement (**a**) for a 400 mg/dL glucose concentration. The dashed line represents the fitted curve of modulation depths to glucose concentrations. (**d**) Clarke’s error grid analysis of the predicted glucose concentration obtained from UOS measurements of glucose solutions. 1 K = 1,000; *UOS* ultrasound-modulated optical sensing, *M*_*d*_ modulation depth, *T*_*d*_ diffuse transmittance.

To demonstrate the feasibility of glucose sensing using the developed UOS system, we obtained UOS signals from various glucose concentrations in a vessel-mimicking phantom. Ultrasound bursts with 10% duty cycle were used to achieve high SNR and prevent the phantom from heating up. Figure 4a shows the normalized modulation depths and diffuse transmittances extracted from the UOS and the conventional diffuse optical sensing results, respectively, and Fig. 4c is a close-up of the measurements within a range of 0–400 mg/dL. The error bars represent ± 1 standard deviation of 20 measurements. The dashed lines and the shaded areas indicate curves of normalized signal fitted to glucose concentration and their 95% confidence intervals. We can clearly see that modulation depth decreases dramatically as glucose concentration increases, but there is no noticeable dependency between diffuse transmittance and glucose concentration in the biologically relevant range (Fig. 4c). Figure 4b shows the predicted glucose concentration of an additional five measurements for each glucose concentration, using the calibration result in Fig. 4a. Figure 4d shows the Clarke’s error grid analysis^52^ of the predicted glucose concentration obtained from the additional five UOS measurements for each glucose concentration, and the calibration results are in Fig. 4c. The prediction accuracy is assessed as 26.6% mean absolute relative difference (MARD), defined by the arithmetic mean of relative absolute differences as follows^53–55^:

3 $$MARD [\%]= \frac{1}{N}\sum_{i=1}^{N}\frac{\left|{y}_{est, i}-{y}_{ref, i}\right|}{{y}_{ref, i}}\times 100,$$

where $N$ is the total number of measurements and ${y}_{est, i}$ and ${y}_{ref, i}$ are the estimated and reference values at the $i$-th measurement, respectively. In the assessment, only data in the range 0–400 mg/dL are used. Note that the FDA guidance for the MARD of a glucose meter is within 20%. Among 45 measurements, 32 predictions fall into zone A, which implies the prediction has a reliable clinical accuracy and can make correct clinical decisions for treatment. However, outliers are observed: five measurements at 50 mg/dL occur in zone D and eight at 100 and 150 mg/dL occur in zone B. The tubing in the phantom might cause a nonlinear effect on light fluence and ultrasonic modulation efficiency, which could lead to a non-negligible estimation error. Exacting preparation of low concentration glucose solutions is also needed. Still, the results show the great potential of the UOS technique for extension to provide absolute glucose concentration values in a noninvasive, agent-free, and real-time manner.

## Discussion

This study has demonstrated the feasibility of a UOS glucose meter in a vessel-mimicking phantom. To achieve strong glucose absorption contrast, we used a 1645 nm laser based on the measured absorbance spectra. This wavelength lies in the first overtone band (1,500–1,800 nm) known to have primary absorption peaks of glucose in the NIR region^56,57^. Two different ultrasound transmission modes, bursts with a 10% duty cycle and continuous waves, were used to modulate diffusive photons in a 20-mm-thick phantom, and the resultant modulation depths were compared. The sensing region was targeted at the middle of the 20-mm-thick phantom, i.e. at 10 mm depth, taking into account the typical depth of the subcutaneous tissues (2–30 mm) where blood glucose is desired to be measured^58,59^. Within the diagnostic ultrasound safety limit, modulation depth in burst mode was improved by 2.5 times over CW mode. We could apply much higher acoustic pressure to the sample in burst mode since the sample could cool during the off cycle, whereas it remained heated in CW mode. The predicted glucose concentration from UOS measurements had a 26.6% MARD accuracy, which demonstrates the feasible use of the device as a noninvasive glucose meter.

The measured absorbance values of glucose solution and heavy water at 1645 nm were 0.523 and 0.264, respectively, which can be converted to absorption coefficients of 1.20 cm^−1^ and 0.61 cm^−1^, respectively. The absorption coefficients of oxyhemoglobin, deoxyhemoglobin, and lipid at 1,600 nm are about 0.9 cm^−1^, 0.8 cm^−1^, and 0.25 cm^−1^, respectively^60^, all of which are lower than the measured absorption coefficient of glucose solution. In the wavelength region of interest in this study, optical absorption in biological tissues is mainly attributed to water^60–64^. In this regard, the feasibility of the proposed technique as a blood glucose sensor was first demonstrated based on the absorbance of glucose solution in water higher than the absorbance of water. Indeed, the absorbance of glucose dissolved in deionized water (0.3 [a.u.]) was higher than that of deionized water (0.06 [a.u.]) at 1645 nm^65^. Even the absorbance of blood at 1645 nm increased as blood glucose level increased within the biologically relevant range^66^.

The effective attenuation coefficient of the phantom was measured as 3.1 cm^−1^, which is slightly lower than that of human tissue, about 12 cm^−1^ at 1645 nm^67,68^. To assess whether the proposed system would be feasible in humans, we estimated the expected maximum penetration depth in human tissue. Assume that the minimum detectable power (${P}_{min}$) of the proposed system is three times NEP (noise equivalent power) of the photodiode, which is calculated as

$${P}_{min}=3\times NEP=3\times \left(1.01\times {10}^{-11}W/\sqrt{Hz}\right)\times \sqrt{\frac{3\times {10}^{8}m/s}{1645\times {10}^{-9}m}}\approx 0.4 mW.$$

With the output power (${P}_{0}$) of the laser, 250 mW, the power ($P$) after penetrating human tissue with a depth of $z$ and the maximum penetration depth (${z}_{MAX}$) can be calculated as follows.

$$P={P}_{0}\times exp\left(-{\mu }_{eff}\times z\right)\ge {P}_{min}$$

$${z}_{MAX}=\frac{ln\left({P}_{0}/{P}_{min}\right)}{{\mu }_{eff}}=\frac{ln\left(250/0.4\right)}{12{cm}^{-1}}\approx 0.54 cm.$$

The maximum penetration depth in human tissue is estimated as 0.54 cm, which is suitable for our target depth. This could be more increased by using a high-sensitive photodetector (e.g. avalanche photodiode) or a high power laser. Note that we used laser power less than 3% of ANSI safety limit in this study.

Thanks to the fact that ultrasound scatters much less than light does in biological tissue, diffusive photons could be localized by ultrasonic modulation and optical properties could be sensed at much higher spatial resolution than with conventional pure optical sensing techniques. Taking this advantage, the proposed technique could be a promising candidate for sensing blood glucose levels in a deep dermal vascular plexus rather than sensing interstitial fluid glucose levels in superficial layers which most noninvasive glucose monitoring techniques are targeted at. Photoacoustic sensing also can provide similar advantages, but requires an expensive and bulky pulsed laser, whereas the proposed UOS system utilizes a simple CW laser and a single element ultrasound transducer, which can easily be miniaturized at low cost and be an excellent candidate for a noninvasive homecare monitoring device.

For successful in vivo application, several challenges are needed to be overcome. (1) This preliminary study implemented the UOS system in transmission mode, which detects diffuse transmittance through scattering phantoms. By modifying the system to work in reflection mode, the technique could be extended to monitoring blood glucose levels in vivo. (2) Pulsatile movement might affect UOS measurements. Since UOS acquisition time in this study (5 ms) is significantly shorter than pulsation repetition period (0.6–1.0 s), we can reduce, or even eliminate the effect by synchronizing UOS measurements with pulsation repetition period. ECG signal can be used for synchronization, or ultrasound itself can be used by exploiting Doppler ultrasonography. (3) Absorption of aqueous glucose could be affected by temperature at the site of measurement. Using the calibration curve for changes in absorbance over temperature^69^, the error due to temperature dependence could be corrected. Thus, further studies incorporating ergonomic design, human blood sample tests, in vivo trials, and calibration for individual subject would be desirable. Even though our study is limited to phantom, we believe that our results proved the feasibility of the proposed technique as a noninvasive glucose meter.

## Methods

### Optical absorption contrast of glucose solution

A UV–Vis–NIR spectrophotometer (Cary 5000, Varian, Australia) was used to obtain the absorbance spectra of glucose solutions at various concentrations. The highest concentration of the glucose solutions was 9,333 mg/dL, achieved by dissolving dextrose powder (D9434, Sigma-Aldrich, USA) in heavy water, and was diluted to various concentrations down to 1% (93 mg/dL). The absorbance spectrum of heavy water, denoted by 0 mg/dL, was also obtained as a control. Each sample was separately placed in a quartz cuvette with a 1 cm light path ($l$), and their transmittance was measured over a wavelength range of 500–2,000 nm, with a 1 nm step size. The absorbance ($A$) was converted from the measured percent transmittance ($\%T$) using the equation $A= 2-log\left(\%T\right)$, and absorption coefficient can be calculated as ${\mu }_{a}=ln\left(10\right)\times A / l$.

### In vitro phantom preparation

**Figure 5:**
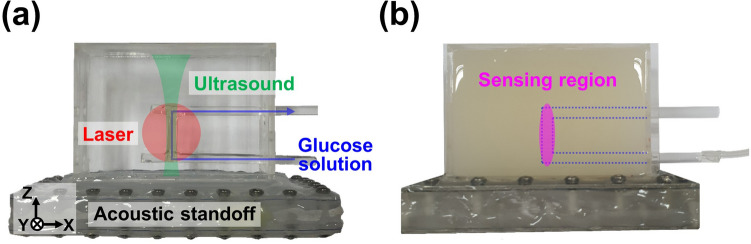
Photographs of a vessel-mimicking phantom with an empty (**a**) and filled (**b**) gelatin-TiO_2_ background embedding a tubing. The red and green areas indicate the diffused light and ultrasound propagation, respectively. The blue arrows and dotted area indicate the pathway of the glucose solution. The magenta area indicates the localized sensing region.

A mold was custom-designed with dimensions of 60 mm (X) $\times$ 20 mm (Y) $\times$ 40 mm (Z) using 1-mm-thick acryl. A lumen tubing (72D Pebax Tubing, Duke Extrusion, USA; 0.156 inch outer diameter; 0.005 inch nominal wall thickness) was positioned in the middle of the phantom mold with respect to the light propagation path (Y) and parallel to the X–Z plane (Fig. 5a). The overlap between the diffused light zone (red-filled area in Fig. 5a) and the ultrasound propagation zone (green-filled area in Fig. 5a) was the localized sensing region. The overlap zone measured about ø4 mm (X–Y plane) $\times$ 15 mm (Z) (magenta-filled area in Fig. 5b). To mimic soft tissues, 0.1 mg/mL TiO_2_ powder (232033, Sigma-Aldrich, USA) was dispersed in heavy water (151882, Sigma-Aldrich, USA) and then 0.2 g/mL gelatin was dissolved in the mixture. The gelatin-TiO_2_ mixture was poured into the mold and solidified with an acrylic lid closed. To increase the efficiency of ultrasound delivery, an acoustic standoff was designed using 3D design software (Inventor, Autodesk, USA) and printed using a 3D printer (ProJet MJP 2500, 3D Systems, USA). The top and the bottom of the acoustic standoff were covered with a thin polycarbonate film, and the inside was filled with water for acoustic coupling.

### In vitro vessel-mimicking phantom measurements

To minimize errors, we first prepared glucose solution with a concentration of 10,000 mg/dL and then diluted the solution in 1,000 mg/dL intervals from 1,000 to 9,000 mg/dL, and in 50 mg/dL intervals from 0 to 400 mg/dL. Each solution was then injected into the tubing (blue dotted area in Fig. 5b) and its UOS signal was measured with an optical wavelength of 1,645 nm. We used 10 ultrasound bursts with a carrier frequency of 1 MHz and a repetition period of 100 μs. The signals were averaged over 50 periods (100 μs/period $\times$ 50 periods = 5 ms).
